# The impact of the comorbid seizure/epilepsy on the health related quality of life in people with multiple sclerosis: an international multicentric study

**DOI:** 10.3389/fimmu.2023.1284031

**Published:** 2023-11-07

**Authors:** Jelena Drulovic, Tatjana Pekmezovic, Olivera Tamas, Ivan Adamec, Dejan Aleksic, Marko Andabaka, Vanja Basic Kes, Silva Butkovic Soldo, Mirjana Cukic, Livija Despinic, Evica Dincic, Jasminka Djelilovic Vranic, Sanja Grgic, Mario Habek, Sonya Ivanova Hristova, Jovana Ivanovic, Aleksa Jovanovic, Vanja Jovicevic, Magdalena Krbot Skoric, Igor Kuzmanovski, Gorica Maric, Sarlota Mesaros, Ivan Gospodinov Milanov, Svetlana Miletic Drakulic, Osman Sinanovic, Ingrid Skarpa Prpic, Josip Sremec, Daliborka Tadic, Gordana Toncev, Dragoslav Sokic

**Affiliations:** ^1^ Clinic of Neurology, University Clinical Center of Serbia, Belgrade, Serbia; ^2^ Faculty of Medicine, University of Belgrade, Belgrade, Serbia; ^3^ Institute of Epidemiology, Faculty of Medicine, University of Belgrade, Belgrade, Serbia; ^4^ Department of Neurology, University Hospital Center of Zagreb, Zagreb, Croatia; ^5^ Department of Neurology, Kragujevac Clinical Center, Faculty of Medical Sciences, University of Kragujevac, Kragujevac, Serbia; ^6^ Department of Neurology, Sestre milosrdnice University Hospital Center, Zagreb, Croatia; ^7^ Department of Neurology, Osijek University Hospital Centre, Faculty of Medicine, Josip Juraj Strossmayer University of Osijek, Osijek, Croatia; ^8^ Department of Neurology, Clinical Center of Montenegro, Podgorica, Montenegro; ^9^ Department of Neurology, General Hospital Subotica, Subotica, Serbia; ^10^ Department of Neurology, Military Medical Academy, Medical Faculty, Defense University, Belgrade, Serbia; ^11^ Clinic of Neurology, Clinical Center University of Sarajevo, Sarajevo, Bosnia and Herzegovina; ^12^ Clinic of Neurology, University Clinical Center of the Republika Srpska, Banja Luka, Bosnia and Herzegovina; ^13^ School of Medicine, University of Zagreb, Zagreb, Croatia; ^14^ Department of Neurology, University Neurological Hospital St Naum, Medical University Sofia, Sofia, Bulgaria; ^15^ Faculty of Electrical Engineering and Computing, University of Zagreb, Zagreb, Croatia; ^16^ University Clinic of Neurology, Clinical Center ‘‘Mother Teresa’’, Faculty of Medicine, Ss Cyril and Methodius University of Skopje, Skopje, North Macedonia; ^17^ Department of Neurology, University Clinical Center Tuzla, Medical Faculty University of Tuzla, Tuzla, Bosnia and Herzegovina; ^18^ Clinic of Neurology, Clinical Hospital Center Rijeka, Faculty of Medicine, University of Rijeka, Rijeka, Croatia; ^19^ Clinic of Neurology, Clinical Hospital ‘‘Sveti Duh’’, Zagreb, Croatia

**Keywords:** multiple sclerosis, seizure, epilepsy, comorbidity, quality of life, depression

## Abstract

**Introduction:**

The health-related quality of life (HRQoL) of people with (Pw) multiple sclerosis (MS) is usually deteriorated. It has been recently suggested that comorbidities may have the negative influence on the quality of life of the PwMS, but according to the best of our knowledge, only one study investigated, although in a very small cohort, the impact of individual comorbidity on the quality of life of PwMS. The aim of our investigation was to assess, in an international, multicentric study, the impact of comorbid seizure/epilepsy on the HRQoL in PwMS.

**Methods:**

We conducted cross-sectional study at numerous neurological centers in Serbia, Croatia, Bulgaria, Montenegro, Northern Macedonia, and Bosnia and Herzegovina (Federation of Bosnia and Herzegovina and Republic of Srpska). For each patient, demographic and clinical data were collected, including Expanded disability status scale (EDSS) score. Beck Depression Inventory (BDI) and the 36-Item Short Form Health Survey (SF-36) questionnaires were administered to all patients.

**Results:**

The study comprised 326 PwMS in total, 127 PwMS with seizure/epilepsy and 209 PwMS without. Both mean Physical health composite (PHC) and mental health composite (MHC) scores, were statistically significantly higher in PwMS without seizure/epilepsy, implicating worse quality of life in PwMS with comorbid seizure/epilepsy. Presence of seizure/epilepsy in pwMS was statistically significant independent predictor of both PHC and MHC, in multivariate linear regression model after adjustment for potential confounding variables. The hierarchical multivariate regression analysis was performed in order to establish the most important predictors of the PHC and MHC of the SF-36, in PwMS with seizure/epilepsy; older age, higher level of disability, as measured by EDSS, higher depression score, drug-resistant epilepsy and shorter time since last seizure were found to significantly predict worse MHC score in PwMS with seizure/epilepsy.

**Discussion:**

Our results point to the possible role of theinterventions related to the adequate control of epilepsy along with improvement of the mental health status to be important in order to reduce MS burden in the PwMS with comorbid seizure/epilepsy.

## 1 Introduction

The multiple sclerosis (MS) International Federation describes the health-related quality of life (HRQoL) of people with (Pw) MS as complex, significantly disturbed and affected by many various factors (1). Numerous studies have already demonstrated that demographic characteristics, disability and its progression, depression, and social status, can significantly decrease the HRQoL of PwMS (2–7). Additionally, since it has been generally accepted that comorbidities, being highly prevalent in PwMS and compelling in the development of the disability progression and mortality in these patients, it might implicate their additional, potential impact on the HRQoL (8, 9). The prevalence of comorbidities in PwMS is highly variable, depending on the number and type of diseases taken into account and the characteristics of the study population (10). It is also partly due to the fact that the data available exist only for certain regions in the world, especially those in Europe and Northern America, and additionally, these findings are inconsistent (11). All of the above also applies to the significant variations in the prevalence of epilepsy, as a comorbidity of MS, reported, until now (10, 12–14). However, it has been generally accepted that the mean prevalence of epilepsy in PwMS is higher than in the general adult population (10, 13).

The impact of group and individual comorbidities on the HRQoL is not clearly defined yet, but existing studies suggest that certain comorbidities worsen outcomes in PwMS (9, 15). Therefore, it may be crucial to analyze individual comorbidities that might affect the HRQoL of PwMS, in order to consequently target them for early detection, and optimal intervention. According to the best of our knowledge, only one study (4) investigated, although in a very small cohort, the impact of individual comorbidity on the quality of life of PwMS. Authors demonstrated, using the MSQoL-54 questionnaire, that comorbid migraine had no influence on the Physical and the Mental composite scores (4). However, the scores, in certain subdomains such as, Role limitation physical, Bodily pain and Health perception, were worse in PwMS with migraine than in those without.

The aim of our investigation was to assess, in the international, multicentric, cross-sectional study, the impact of comorbid seizure/epilepsy on the HRQoL in PwMS.

## 2 Methods

We conducted an international, multicentric, cross-sectional study at numerous neurological centers in Serbia, Croatia, Bulgaria, Montenegro, North Macedonia, and Bosnia and Herzegovina. Patients who were considered to participate in this study were PwMS in whom the diagnosis was established according to the Revised McDonald criteria 2017 (16) with comorbid seizure/epilepsy. The diagnosis of epilepsy was established according to the first criteria of the ILAE practical clinical definition of epilepsy (“At least two unprovoked, or reflex, seizures occurring >24 h apart”) (17). Control group comprised comparable number of sex/age grouped matched PwMS, free from seizure/epilepsy, from all participating centers. Since patients in two groups should have been sex- and age-matched, in all centers, for each pwMS with seizure/epilepsy, the first consecutive sex- and age-matched pwMS without seizure/epilepsy were recruited. For each patient, demographic and clinical data were collected, including the Expanded disability status scale (EDSS) score (18). The disease duration referred to the period from the onset of neurological manifestations of MS until last visit. Beck Depression Inventory (BDI) (19) and a generic quality of life questionnaire, the 36-Item Short Form Health Survey (SF-36) (20, 21), were administered to all patients. The SF-36 is a generic questionnaire for the assessment of the HRQoL in eight domains: physical functioning (PF), role functioning physical (RP), bodily pain (BP), general health (GH), energy (E), social functioning (SF), role functioning emotional (RE), and mental health (MH). Two summary scales were composed comprising these eight domains: the Physical Health Composite (PHC) and the Mental Health Composite (MHC). Scoring and the calculations were performed by using the original Ware’s method (21). Quality of life scores were presented as T scores (mean = 50; SD = 10), which were obtained by linear transformation of raw scores, that facilitates comparisons across the multiple subscales of the SF-36 (21). The items are summed, thus defining scores which range from 0 (worst possible health state) to 100 (best possible health state). Having that in mind, higher values denote better functioning and well-being. The BDI was completed by each patient in order to assess their individual feelings and attitudes which are believed to express their general depressive state. Higher scores of BDI indicate more severe depressive symptoms (19).

For Pw MS and comorbid seizure/epilepsy, all relevant epilepsy-related information, such as: type of epilepsy, frequency of seizures, time since last seizure, occurrence of status epilepticus, anti-seizure medications (ASM) administered, and the presence of the drug-resistant (DR) epilepsy, based on the assessment of the epilepsy specialist using patient medical history and medical check up findings were collected. DR epilepsy was considered in persons who failed to be seizure free with trials of at least two adequately applied ASMs. Subjects, who were unable to provide personal data or refused to participate in the study, and those with intellectual disability or severe mental disorders, were excluded.

The study was approved by the Ethical Committee of the Faculty of Medicine University of Belgrade (no. 29/III-20). All participants signed inform consent prior to the study inclusion. The study followed the Declaration of Helsinki and the current European Regulation for Data Protection.

### 2.1 Statistical analysis

The differences in the demographic and clinical characteristics between PwMS with seizure/epilepsy and those without this comorbidity, were compared using chi-square test for categorical variables, t-test for continuous variables with normal distribution, and Mann-Whitney U test for non-normally distributed variables. In order to assess the independent contribution of seizure/epilepsy comorbidity on HRQoL of PwMS, a multivariate linear regression model was applied, which included demographic and clinical variables as potential confounding factors. The hierarchical multiple regression analysis was conducted to identify predictors of the PHC and MHC scores of the SF-36, as outcome variables. Predictor variables were separated into three blocks (models). Age was entered in the first block, clinical variables (EDSS and BDI scores) comprised the second block, and epilepsy-related characteristics, separately, in the third block. The level of statistical significance was set at p<0.05. All analyses were performed using the Statistical Package for Social Sciences (SPSS), version 17.0.

## 3 Results

A total of 326 subjects, 127 PwMS with seizure/epilepsy and 209 PwMS without, were enrolled in the study. In our cohort, 109 participants fulfilled the criteria of the ILAE (17) for the diagnosis of epilepsy, while the remaining 18 had only one unprovoked, or reflex, seizure. Their demographic and clinical characteristics are presented in **Table 1**. There were no differences between MS patients with and without seizure/epilepsy, except for the marital status (p=0.001). PwMS with seizure/epilepsy were statistically significantly more frequently unmarried. As presented in the **Table 1**, there was no significant difference in the proportion of MS patients treated with DMTs. Various molecules have been used in both groups (interferon beta preparations, glatiramer acetate, teriflunomide, dimethyl fumarate, fingolimod, cladribine tabl, alemtuzumab, daclizumab, and ocrelizumab).

**Table 1:** Demographic and clinical characteristics of the study subjects.

Variable	MS with epilepsy (n=127)	MS without epilepsy (n=209)	p
Gender (n, %) -male -female	38 (29.9) 89 (70.1)	49 (23.4) 160 (76.6)	0.189
Age (mean ± SD) (years)	40.6 ± 11.9	42.4 ±11.1	0.162
Marital status (n, %) -married -unmarried	53 (43.1) 70 (56.9)	136 (65.4) 72 (34.6)	0.001
Education (years)	12.9 ± 3.1	13.2 ± 2.5	0.276
MS phenotype (n, %) -RRMS -SPMS -PPMS	88 (71.0) 23 (18.5) 13 (10.5)	155 (74.2) 29 (13.9) 22 (10.5)	0.259
EDSS (median, IQR)	3.0 (4.5)	3.0 (3.5)	0.646
DMT (n, %) -yes -no	52 (43.0) 69 (57.0)	109 (52.4) 99 (47.6)	0.099

MS-Multiple sclerosis; RRMS-relapsing-remitting MS; SPMS-secondary progressive MS; PPMS, primary progressive MS; EDSS, Expanded disability status scale; IQR, interquartile range;

DMT, disease modifying treatment.

The median duration of epilepsy was 5.0 years (IQR-interquartile range, 13). Twenty three (18.1%) experienced status epilepticus during the course of the disease. Six subjects (4.8%) had seizure frequency higher than once per month, and 26 (20.5%) had last seizure during the last 12 months. According to the assessment of epilepsy specialist, 22 (17.3%) of the people had focal onset epilepsy, 30 (23.6%) had focal onset to bilateral tonic-clonic seizure, and 43 (33.9%) had generalized onset. A total of 109 (85.8%) patients took old ASMs (valproic acid, clonazepam, phenobarbital, carbamazepine, oxcarbazepine), 60 (47.2%) new ASMs (levetiracetam, pregabalin, topiramate, lamotrigine), and 21 (16.5%) were treated with at least two ASMs. DR epilepsy had 25 (19.7%) subjects.

Mean scores of all SF-36 domains were significantly lower in PwMS with seizure/epilepsy in comparison with PwMS without seizure/epilepsy, except the Emotional Well-Being (EWB) which was also higher in PwMS without seizure/epilepsy, but the difference did not reach statistical significance (p=0.054) (**Table 2**). Also, both mean PHC and MHC scores, were statistically significantly higher in PwMS without seizure/epilepsy, implicating worse quality of life in PwMS with comorbid seizure/epilepsy (**Table 2**). Mean BDI score was statistically significantly higher in PwMS with comorbid seizure/epilepsy, demonstrating higher level of depression in those with comorbidity (p=0.002).

**Table 2:** Description of the SF-36 and BDI scores in MS patients with and without seizure/epilepsy.

	MS with seizure/epilepsy (n=127)	MS without seizure/epilepsy (n=209)	p
Scale	Score (mean ± SD)		
SF-36 domains			
- Physical Health	57.1 ± 34.9	65.9 ± 30.3	**0.016**
- Physical Role Limitations	34.6 ± 42.2	52.3 ± 43.3	**< 0.001**
- Emotional Role Limitations	44.2 ± 44.7	56.8 ± 43.9	**0.012**
- Energy	50.3 ± 23.7	57.6 ± 24.1	**0.008**
- Emotional Well-Being	62.5 ± 23.6	67.6 ± 23.6	0.054
- Social Function	59.6 ± 28.9	69.9 ± 25.5	**0.001**
- Bodily pain	62.7 ± 30.0	70.1 ± 26.5	**0.020**
- General Health	48.1 ± 23.2	55.0 ± 23.0	**0.009**
Physical Health Composite	50.6 ± 26.0	60.8 ± 25.9	**0.001**
Mental Health Composite	54.1 ± 24.9	62.9 ± 24.7	**0.002**
BDI	10.1 ± 9.1	7.2 ± 7.3	**0.002**

MS-Multiple sclerosis; SD-standard deviation; BDI-Beck Depression Inventory;

Bold values denote statistical significance.

In order to assess the independent contribution of seizure/epilepsy comorbidity on HRQoL of PwMS, a multivariate linear regression model was applied, which included demographic (gender, age) and clinical variables (disease duration, MS phenotype, treatment with DMTs, EDSS, and BDI scores) as potential confounding factors. Presence of seizure/epilepsy in pwMS was statistically significant independent predictor of both, PHC and MHC, in multivariate linear regression model after adjustment for all above mentioned confounding variables (**Table 3**).

**Table 3:** Impact of seizure/epilepsy comorbidity on HRQOL of MS patients, assessed by a multivariate linear regression analysis.

	Physical Health Composite			Mental Health Composite		
Variables	B	95% CI for B	p	B	95% CI for B	p
- Seizure/epilepsy comorbidity	9.56	4.81,14.31	**<0.001**	5.03	0.73, 9.32	**0.022**
- Age	-0.74	-0.94, -0.54	**<0.001**	-0.39	-0.57, -0.21	**<0.001**
- EDSS score	-0.25	-0.45, -0.05	**0.016**	-0.08	-0.27, 0.12	0.458
- BDI score	-1.57	-1.85, -1.29	**<0.001**	-1.96	-2.22, -1.70	**<0.001**

MS-Multiple sclerosis; EDSS-Expanded Disability Status Scale, BDI-Beck Depression Inventory; B-beta coefficient, 95% CI-confidence interval, Bold values denote statistical significance.

The hierarchical multivariate regression analysis was performed in order to establish the most important seizure/epilepsy-related predictors of the PHC and MHC of the SF-36, in PwMS with seizure/epilepsy. Using the MHC score as a dependent variable, the first block consisting of age accounted for 7.7% of the variance in the outcome variable (p<0.01) (**Table 4A**). Addition of clinical variables (EDSS and BDI scores) in the second block, led to an increase of 48.2% in the variance explained (p<0.01). After adding the variable DR epilepsy in the third block, an additional 3.4% of the variance in the outcome variable has been explained (p<0.05). Similarly, **Table 4B** shows that age in the first block accounted for 13.7% of the variance in the outcome variable (p<0.01), EDSS and BDI scores in the second block caused an increase of 50.0% in the variance explained (p<0.001), and adding the time since last seizure in the third block, an additional 3.9% of the variance in outcome variable has been explained (p<0.05). None of the remaining epilepsy-related information was found to be predictor of the MHC score.

**Table 4A:** Summary of hierarchical regression analysis for variables predicting Mental Health Composite Score of the SF-36: the predictive effect of the drug-resistant epilepsy.

Variable	Model 1			Model 2			Model 3		
	B	SE (B)	β	B	SE (B)	β	B	SE (B)	β
***Age***	-0.64	0.22	-0.28^**^	-0.05	0.20	-0.02	-0.08	0.20	-0.03
Clinical variables									
EDSS score				-2.23	0.91	-0.21^*^	-2.02	0.89	-0.19^*^
BDI score				-1.57	0.20 -	0.59^**^	-1.60	0.20	-0.61^**^
***Drug-resistant epilepsy***							16.16	6.20	0.19^*^
**R^2^**		0.077			0.482			0.516	
**F for change in R^2^**		8.300^**^			38.273^**^			6.795^*^	

^*^p<0.05, ^**^p<0.01, B-unstandardized beta coefficient, SE-standard error, β-standardized beta coefficient, R^2^-coefficient of determination.

**Table 4B:** Summary of hierarchical regression analysis for variables predicting Mental Health Composite Score of the SF-36: the predictive effect of the time since last seizure.

	Model 1			Model 2			Model 3		
Variable	B	SE (B)	β	B	SE (B)	β	B	SE (B)	β
***Age***	-0.90	0.25	-0.37^**^	-0.14	0.23	-0.06	-0.27	0.23	-0.06
Clinical variables									
EDSS score				-2.75	1.03	-0.25^**^	-2.31	1.01	-0.21^*^
BDI score				-1.57	0.23	-0.57^**^	-1.59	0.22	-0.58^**^
***Time since last seizure***							10.52	4.02	0.20^*^
**R^2^**		0.137			0.500			0.539	
**F for change in R^2^**		13.215^**^			29.356^**^			6.835^*^	

^*^p<0.05, ^**^p<0.01, B-unstandardized beta coefficient, SE-standard error, β-standardized beta coefficient, R^2^-coefficient of determination.

When using the PHC score as a dependent variable, none of the variables related to epilepsy, tested in the third block, was found to be significant predictor of the PHC score.

## 4 Discussion

Our study shows that the presence of comorbid seizure/epilepsy, as an independent predictor of the HRQoL in pwMS, statistically significantly negatively affects the quality of life, as demonstrated by both PHC and MHC scores. Furthermore, mean scores of all SF-36 domains were significantly lower in PwMS with seizure/epilepsy in comparison with PwMS without seizure/epilepsy, except the EWB. The deleterious effect of this comorbidity on the quality of life of PwMS is not surprising. It is well known that the quality of life of people with epilepsy is significantly disturbed (22, 23). It has been shown that epilepsy-related factors have limited influence on the quality of life of patients with epilepsy and that, on the other hand, mood disorders (anxiety and depression) have the most significant impact in these patients (23, 24). However, considering the influence of epilepsy-related factors, it has to be emphasized that some of them also affect quality of life. Thus, it was shown that high seizure frequency might be associated with poorer quality of life (23, 25), although these results are not fully consistent (26). It has been also shown that the predictor of lower quality of life was occurrence of seizures, the week before the interview (27). Furthermore, since a number of patients remain resistant to ASMs, it has been shown that the overall quality of life scores were statistically significantly higher in the group of patients who were responders as compared to non-responders (28). In line with these findings, it has been also, recently, shown that the insufficient seizure control with high frequency of seizures is significantly related to the poor quality of life (25).

In our investigation, dealing with factors potentially predicting quality of life in PwMS with seizure/epilepsy, the outcome variables were the composite scores of SF-36. Predictive variables included demographic characteristics (age), MS-related variables (EDSS and BDI scores), and seizure/epilepsy-related variables (DR epilepsy and time since last seizure). In the first step, in both models, with MHC as a dependent variable, age was the strong demographic predictor of quality of life (p<0.001). After adding MS-related variables and seizure/epilepsy related variables, age, was no longer significant. In the second step, EDSS and BDI scores, were entered as predictors, and the total variance explained were 48.2% (p<0.001) and 50.0% (p<0.001). The results showed that the disability (p<0.05) and depression (p<0.001) had significant detrimental effect on the MHC. In the third step, DR epilepsy and time since last seizure were entered as predictors in two models, respectively, and the total variance explained by the models was 3.4% (p<0.05) and 3.9% (p<0.05), respectively. Thus, older age, higher level of disability as measured by EDSS, higher depression scores, presence of DR epilepsy and shorter time since last seizure were found to significantly contribute to worse quality of life in PwMS with seizure/epilepsy. Similar concept using hierarchical multivariate regression analysis performed in order to assess predictors of quality of life in patients with epilepsy was recently reported (29). Authors have demonstrated, in line with our findings, that lower depression scores were found to contribute significantly to higher overall quality of life. Additionally, they have shown that higher acceptance of disability and higher social support also have beneficial effect on quality of life of these patients.

According to our findings, it may be important to consider focus on attaining seizure freedom in PwMS with seizure/epilepsy using appropriate ASMs, in order to improve their quality of life, although the impact of these factors on the outcome is relatively limited. However, on the other hand, emotional disorder has been confirmed to be an important predictor of the worse quality of life of PwMS and comorbid seizure/epilepsy. This notion is in line with findings showing that depressive mood disorders are common comorbidity and represent well known risk factor for reduced quality of life in patients with epilepsy (22–25, 29). Therefore, it is not unexpected that we found mean depressive scores being higher in PwMS and comorbid seizure/epilepsy in comparison to PwMS without seizure/epilepsy. This might be one of the reasons that in our cohort, mean quality of life scores were worse in PwMS with comorbid seizure/epilepsy. Comorbidities are important for clinicians treating PwMS because some of them are rather common and potentially preventable or treatable. It has been suggested that comorbidities may have the negative influence on the quality of life of the PwMS, this notion being increasingly reported in the last years (2, 4, 9). Recently, cross-sectional study comprising 902 Australian MS longitudinal study participants, examined the magnitude of the effects of individual comorbidities on the HRQoL (9). It has been shown that comorbidities accounted for about 18% of the variance in the HRQoL. Among all comorbidity groups, mental health contributed most to the overall HRQoL with even about 68% of the overall comorbidity effect (9). Even higher contribution was demonstrated for the psychosocial super-dimension, being more than 80% (9). Additionally, it has to be stressed that among individual comorbidities, systemic lupus erythematosus and depression were most strongly associated with total HRQoL. Our data support these findings, consequently suggesting that management of the comorbidities has significant potential to beneficially influence the HRQoL in PwMS. It may be crucial to present the valuable effects of interventions on patient-reported outcomes because they are related to those which reflect certain issues of utmost importance for patients. Thus, our investigation which assessed the impact of individual comorbidity, seizure/epilepsy on the HRQoL, implicates that it might be considered that the interventions related to adequate control of epilepsy and the improvement of the mental health status are crucial in order to reduce MS burden in the cohorts of PwMS with comorbid seizure/epilepsy.

Our study has certain limitations. Firstly, follow-up studies instead of cross-sectional design are warranted to be performed, in order to confirm obtained findings. Secondly, the selection of the control group, in our study, might be the potential source of the selection bias. Finally, usage of the disease specific HRQoL questionnaires might have been more adequate because they could potentially provide data regarding additional quality of life domains.

## 5 Conclusion

In conclusion, a significant proportion of persons with MS with seizure/epilepsy, have considerably lowered quality of life. Further investigations are necessitated in order to optimize treatment interventions in PwMS with individual comorbidities, including those with MS and comorbid seizure/epilepsy, in order to improve their HRQoL.

## Data availability statement

The raw data supporting the conclusions of this article will be made available by the authors, without undue reservation.

## Ethics statement

The studies involving humans were approved by the Ethical Committee of the Faculty of Medicine University of Belgrade (no. 29/III-20). The studies were conducted in accordance with the local legislation and institutional requirements. The participants provided their written informed consent to participate in this study.

## Author contributions

JD: Conceptualization, Methodology, Writing – original draft, Writing – review & editing. TP: Conceptualization, Formal Analysis, Methodology, Writing – review & editing. OT: Data curation, Investigation, Project administration, Writing – review & editing. IA: Data curation, Investigation, Writing – review & editing. DA: Data curation, Investigation, Writing – review & editing. MA: Data curation, Investigation, Writing – review & editing. VB: Investigation, Supervision, Writing – review & editing. SB: Investigation, Supervision, Writing – review & editing. MC: Investigation, Supervision, Writing – review & editing. LD: Data curation, Investigation, Writing – review & editing. ED: Investigation, Supervision, Writing – review & editing. JD: Investigation, Supervision, Writing – review & editing. SG: Investigation, Supervision, Writing – review & editing. MH: Conceptualization, Investigation, Supervision, Writing – review & editing. SH: Data curation, Investigation, Writing – review & editing. JI: Data curation, Investigation, Writing – review & editing. AJ: Formal Analysis, Writing – review & editing. VJ: Data curation, Investigation, Writing – review & editing. MK: Formal Analysis, Writing – review & editing. IK: Investigation, Supervision, Writing – review & editing. GM: Formal Analysis, Writing – review & editing. SM: Conceptualization, Investigation, Writing – review & editing. IM: Investigation, Supervision, Writing – review & editing. SM: Data curation, Investigation, Writing – review & editing. OS: Investigation, Supervision, Writing – review & editing. IS: Investigation, Supervision, Writing – review & editing. JS: Data curation, Investigation, Writing – review & editing. DT: Data curation, Investigation, Writing – review & editing. GT: Investigation, Supervision, Writing – review & editing. DS: Conceptualization, Supervision, Writing – review & editing.
